# Seroprevalence and Seroconversion of Hepatitis B and C Viruses in Patients Undergoing Hemodialysis: A Cross-sectional Study

**DOI:** 10.7759/cureus.67722

**Published:** 2024-08-25

**Authors:** Vanshaj Tayal, Umesh Hassani, Kalpana P Date, Nandkishor J Bankar, Ashwini A Tidake, Ankit Badge

**Affiliations:** 1 Microbiology, N.K.P. Salve Institute of Medical Sciences and Research Centre, Nagpur, IND; 2 Microbiology, Datta Meghe Medical College, Datta Meghe Institute of Higher Education and Research (DU), Nagpur, IND

**Keywords:** hepatitis c virus (hcv), hepatitis b virus (hbv), hemodialysis, hepatotropic viruses, seroconversion, seroprevalence

## Abstract

Introduction: Among hemodialysis patients, hepatitis B virus (HBV) and hepatitis C virus (HCV) infections contribute significantly to mortality and morbidity. Infection with these hepatotropic viruses in hemodialysis patients is due to their increased contact with blood and its derivatives. Additionally, not following the proper protocol for infection control, contaminated devices, and untrained personnel contribute to the nosocomial transmission of these infections. This cross-sectional study was planned to estimate the seroprevalence of hepatitis B surface antigen (HBsAg) and anti-hepatitis C virus antibodies in patients to know the seroconversion rate for hepatitis B and hepatitis C and to evaluate the risk factors that contribute to seroconversion in patients undergoing hemodialysis at our center.

Materials and methods: This study included 185 patients with chronic kidney disease undergoing hemodialysis in our center. After giving informed consent, a blood sample from each patient was collected for testing for HBsAg and anti-HCV antibodies initially and then every month.

Results: Of the total 185 patients, five participants tested positive for HBV (2.7%), and 29 individuals tested positive for HCV (15.67%). During the study period, seroconversion for hepatitis C was observed in three patients (1.62%), and seroconversion for hepatitis B was observed in one patient (0.54%). An evaluation of the potential risk factors revealed that dialysis conducted outside the facility contributed significantly to HCV infection.

Conclusion: Our study shows lower HBV rates but higher HCV rates. The demographic data of the patients and the duration of dialysis are related to the risk of infection. Dialysis within the same healthcare facilities reduces the transmission risk.

## Introduction

Hepatitis B virus (HBV) and hepatitis C virus (HCV) infections pose a significant challenge in underdeveloped countries and contribute significantly to liver diseases worldwide. Chronic HBV and HCV infections can progress to life-threatening conditions such as hepatocellular carcinoma and liver cirrhosis [1]. Among hemodialysis patients, HBV and HCV infections contribute significantly to mortality and morbidity [2].

Statistically, in India, approximately "40 million people suffer from hepatitis B, and 6-12 million people are affected by hepatitis C." Globally, these numbers increase to approximately "296 million for hepatitis B, and 58 million for hepatitis C" [3,4]. In regions with a high prevalence of hepatotropic viruses, co-infection with hepatitis B and hepatitis C is common. The contributing factors to the infection with these hepatotropic viruses in hemodialysis patients are elevated contact with blood and its derivatives, immune dysfunction, and compromised renal functions [5]. Furthermore, not following proper infection control protocol, the use of contaminated devices, and untrained personnel can also contribute to the nosocomial transmission of these infections [6].

The prevalence of both these infections in patients undergoing hemodialysis significantly varies between different countries (1%-84.6%) and also varies among dialysis facilities within a single nation [7]. Immunocompromised status may exacerbate infections resulting from hepatitis viruses in patients due to irreversible kidney damage [8]. This study was planned to estimate the seroprevalence of hepatitis B surface antigen (HBsAg) and anti-hepatitis C virus antibodies (anti-HCV), co-infection of HBV and HCV, and risk factors for seroconversion of HBsAg and anti-hepatitis C virus antibodies (anti-HCV) in patients with end-stage renal disease undergoing hemodialysis at our hospital.

## Materials and methods

This cross-sectional study was conducted on patients with chronic kidney disease who undergo hemodialysis at our hospital. The study was carried out in the Microbiology Department within a tertiary care hospital situated in central India.

Participants who met the inclusion criteria were included. The inclusion criteria were all age groups, including pediatric, adult, and elderly patients, who were undergoing hemodialysis for end-stage renal disease at least once a month. Participants with acute kidney injury (AKI) on temporary dialysis, having a known history of hepatitis B or hepatitis C infections, incomplete medical history, and those who were not willing to participate in the study were excluded from the study. This study included 185 participants from various age brackets who underwent hemodialysis for end-stage renal disease between January 2021 and August 2023 at our tertiary care hospital. Each patient was included only once. Demographic information was collected for each patient and their informed consent was obtained.

As part of the standard protocol, at first dialysis and every month thereafter, the status of hepatitis B and C was tested for each patient undergoing hemodialysis. A 5 ml blood sample was collected in a plain vacutainer from each patient. To separate the serum, the blood sample was centrifuged for 10 minutes at 4000 rpm. The serum was stored at -80°C for later use. HBsAg was detected by an enhanced rapid immunochromatographic test, and anti-HCV antibodies were detected by chemiluminescent immunoassay. The tests for both were carried out according to the manufacturer's instructions. In addition to these tests, liver function tests (LFT) and kidney function tests (KFT) were performed on all the patients.

The results were recorded and analyzed in Microsoft Excel (Microsoft, Redmond, WA) for frequency distribution, proportion rates, and ratios. The data was reviewed for any inconsistency or missing values and categorical variables were coded and checked for normality. Frequency distribution was calculated for demographic and clinical variables, and results were presented as counts and percentage. Independent t-test was used to calculate the continuous variables and the comparison between groups (hepatitis positive and negative). A P value < 0.05 was considered statistically significant.

## Results

The study was carried out on 185 participants who underwent hemodialysis for end-stage renal disease (ESRD). The majority of patients 120 (64.86%) were between 41 and 60 years of age, and the participants were predominantly male (141; 76.22%) (Table 1).

**Table 1:** Demographic dissemination of hemodialysis patients

Age (in years)	Males (%)	Females (%)	Total (%)
<20	2 (1.08%)	0 (0%)	2 (1.08%)
21-40	22 (11.89%)	4 (2.16%)	26 (14.05%)
41-60	85 (45.95%)	35 (18.92%)	120 (64.86%)
>60	32 (17.30%)	5 (2.70%)	37 (20%)
Total	141 (76.22%)	44 (23.78%)	185 (100%)

Out of the total 185 patients, initially, five participants tested positive for HBV and 29 individuals tested positive for HCV. No patient was co-infected with both viruses. During the study period, seroconversion to hepatitis C was observed in three patients (1.62%) and seroconversion to hepatitis B was observed in one patient (0.54%). The seroprevalence of HBsAg was 2.7% and HCV antibodies were 15.68%. Seropositivity for hepatitis C was highest 15 (51.72%) in the 21- to 40-year age group, and hepatitis B positivity was highest in the >60-year age group (60%) (Table 2).

**Table 2:** Prevalence of hepatitis B and C in different age categories among hemodialysis patients HBV: hepatitis B virus; HCV: hepatitis C virus

Age (in years)	Number of HBV-positive patients (%)	Number of HCV-positive patients (%)	No. of HBV- and HCV-positive patients (co-infection) (%)
<20	0 (0%)	0 (0%)	0 (0%)
21-40	1 (0.54%)	15 (8.11%)	0 (0%)
41-60	1 (0.54%)	6 (3.24%)	0 (0%)
>60	3 (1.62%)	8 (4.32%)	0 (0%)
Total	5 (2.70%)	29 (15.68%)	0 (0%)

Statistical analysis was performed to correlate variables potentially associated with the risk of acquiring HBV and HCV infections. Among the patients who underwent dialysis for one year or less, all patients were seronegative. In the group of patients who underwent hemodialysis for one to two years, 14 individuals (48.27%) tested positive for HCV, while one individual (20%) tested positive for HBV. In patients who underwent dialysis for more than two but less than three years, 12 patients (41.37%) were HCV positive and two patients (40%) were positive for HBV.

Table 3 summarizes the risk factors potentially correlated with HBV infection. The mean age of HBV-positive patients was slightly higher (57.60 ± 7.635) than HBV-negative patients (52.26 ± 11.848), although the difference was not statistically significant (p = 0.318). The mean duration of dialysis was longer in HBV-positive patients (2.20 ± 0.447 years) compared to HBV-negative patients (1.77 ± 1.046 years), but this difference was also not statistically significant (p = 0.364). Neither blood transfusion (p = 0.606) nor receiving dialysis outside the current facility (p = 0.382) showed a significant correlation with HBV infection, indicating these factors may not significantly contribute to HBV risk in this patient population.

**Table 3:** Risk factors that might be correlated with HBV infection p<0.05 means significant, SD: standard deviation; HBV: hepatitis B virus; n, number of patients

Risk factor	HBV positive	HBV negative	p Value
Age (mean ± SD)	57.60 ± 7.635	52.26 ± 11.848	0.318
Duration of dialysis (years) (mean ± SD)	2.20 ± 0.447	1.77 ± 1.046	0.364
Blood transfusion (n, %)	3 (1.62%)	134 (72.43%)	0.606
Dialysis outside (n, %)	4 (2.16%)	98 (52.97%)	0.382

Table 4 presents the risk factors potentially associated with HCV infection. The mean age of HCV-positive patients was slightly lower (50.67 ± 9.550) than that of HCV-negative patients (52.74 ± 12.153), with no statistically significant difference (p = 0.380). The mean duration of dialysis was marginally shorter in HCV-positive patients (1.63 ± 0.928 years) compared to HCV-negative patients (1.81 ± 1.056 years), which was also not statistically significant (p = 0.386). Blood transfusion history did not significantly correlate with HCV infection (p = 0.580). However, receiving dialysis outside the current facility was significantly associated with HCV infection (p = 0.001), indicating a potential risk factor for acquiring HCV among these patients.

**Table 4:** Factors that could be correlated with HCV infection p<0.05 means significant; SD: standard deviation; HCV: hepatitis C virus; n, number of patients

Risk factor	HCV positive	HCV negative	p Value
Age (mean ± SD)	50.67 ± 9.550	52.74 ± 12.153	0.380
Dialysis duration (mean ± SD)	1.63 ± 0.928	1.81 ± 1.056	0.386
Blood transfusion (n,%)	21 (11.35%)	116 (62.7%)	0.580
Dialysis outside (n,%)	25 (13.51%)	77 (41.62%)	0.001

## Discussion

Infection with hepatitis B and C viruses poses a significant health concern among dialysis patients. Despite the implementation of effective infection control protocols aimed at reducing patient-to-patient transmission, sporadic outbreaks still occur within dialysis units, often due to lapses in adherence to these protocols. Furthermore, the increased prevalence of these viruses in specific regions contributes to the infection burden within the dialysis population [1].

In our investigation, a predominant proportion of patients were men aged between 41 and 60 years (constituting 64.86% of the cohort). This finding is consistent with several other studies that similarly report that a majority of patients fall within the 40-60 years age bracket. The average age of the participants in our study was 45.8 years, with an age range of 16-66 years. This higher prevalence among people aged 41-60 years might be correlated with an increased exposure to potential risk factors associated with HBV and HCV co-infection.

In the current observation, out of the total 185 patients, five participants tested positive for HBV (2.7%), and 29 individuals tested positive for HCV (15.67%). No patient was co-infected with both viruses. The seropositivity of HCV was maximum (51.72%) in the 21- to 40-year age group, while hepatitis B positivity was highest in those over 60 years old (60%).

The World Health Organization (WHO) states that among the Southeast Asian countries, India is in the intermediate prevalence zone (2% to 5%) [9]. According to the National Center for Disease Control (NCDC) in India, "there is a reported point prevalence of 3.7% for HBV carriers equal to over 40 million individuals affected in the country. Additionally, the population prevalence of HCV infection in India stands at 1%" [10].

In hemodialysis patients, the prevalence of HBV and HCV is significantly higher compared to the general community. Many studies have reported HBV infection rates ranging from 4% to 11% among hemodialysis patients [11-13]. However, in the present study, the seroprevalence of HBV is lower than the typical range observed in other studies. This discrepancy could be attributed to the widespread availability and effectiveness of vaccination programs for all people undergoing hemodialysis.

In the current study, seroconversion for hepatitis C was observed in three patients (1.62%) and seroconversion for hepatitis B was observed in one patient (0.54%). HCV infection is notably more widespread among hemodialysis patients, exhibiting variations across different countries and even within dialysis units within the same country. The literature search for seroconversion for HCV revealed varying figures, ranging from 2% in developed countries to around 40% in developing and underdeveloped countries [14-16]. Previous investigations focusing on the Indian population have reported HCV infection rates ranging from 8% to 12% [17]. The figures for seroconversion observed in our study are similar to most studies, and this implies good implementation of infection control measures.

In the present study, various potential risk factors (age, duration of dialysis, blood transfusion, and outside dialysis) were considered, and their association with seropositivity for hepatitis B and hepatitis C was calculated. The comparison of the above variables associated with HBV infection among individuals undergoing dialysis revealed no statistically significant difference (p>0.05) for all four risk factors studied. In contrast, observations regarding HCV infection revealed that dialysis conducted outside the facility contributed significantly to HCV infection (p=0.001).

To assess the trends in prevalence, incidence, and risk factors for HCV infection, a large multicentric study, 'Dialysis Outcomes and Practice Patterns Study (DOPPS 1996-2015),' was conducted. The study reported that the high prevalence of HCV in the hemodialysis unit was a powerful facility-level risk factor for seroconversion, which is similar to our study [18].

Limitations

As a single-center study, the results cannot be generalized to other regions or populations. Although the size of the sample is adequate for preliminary assessments, the ability to detect subtler connections or variations is limited. Furthermore, relying on self-reported data on certain variables, such as the history of blood transfusions and transfusions in external facilities, may cause biases in reporting. Future multicentred studies, with larger sample sizes and standardized data collection methods, should validate and expand these findings.

## Conclusions

Hepatitis B and C infections remain common among dialysis patients despite ongoing efforts at infection control. Our study reveals lower HBV rates but higher HCV rates, emphasizing the importance of maintaining strict infection control protocols. Dialysis conducted within healthcare facilities has been shown to reduce the risk of viral transmission. To effectively address infections in this vulnerable population, it is crucial to employ comprehensive strategies and continue research efforts.

## References

[1] Mittal G, Gupta P, Thakuria B, Mukhiya GK, Mittal M (2013). Profile of hepatitis B virus, hepatitis C virus, hepatitis d virus and human immunodeficiency virus infections in hemodialysis patients of a tertiary care hospital in Uttarakhand. J Clin Exp Hepatol.

[2] Malhotra R, Soin D, Grover P, Galhotra S, Khutan H, Kaur N (2016). Hepatitis B virus and hepatitis C virus co-infection in hemodialysis patients: a retrospective study from a tertiary care hospital of North India. J Nat Sci Biol Med.

[3] (2024). Fast facts: Global hepatitis B vaccination. https://www.cdc.gov/global-hepatitis-b-vaccination/data-research/index.html.

[4] (2024). Global viral hepatitis. https://www.cdc.gov/hepatitis/global/index.html.

[5] Khan D, Shakeel HA, Maqsood H, Aziz HM, Qazi MJ, Shah SA (2018). Immunization status of students of Nishtar Medical University against hepatitis B. Int J Res Med Sci.

[6] Sagnelli E, Coppola N, Pisaturo M (2009). HBV superinfection in HCV chronic carriers: a disease that is frequently severe but associated with the eradication of HCV. Hepatology.

[7] Prakash S, Jain A, Sankhwar SN (2014). Prevalence of hepatitis B & C viruses among patients on hemodialysis in Lucknow, Uttar Pradesh. Clin Epidemiol Glob Health.

[8] Bhaumik P, Debnath K (2012). Prevalence of hepatitis B and C among haemodialysis patients of Tripura, India. Euroasian J Hepato-Gastroenterol.

[9] (2024). Hepatitis B. https://www.who.int/news-room/fact-sheets/detail/hepatitis-b.

[10] (2024). Viral hepatitis - the silent disease facts and treatment guidelines. https://ncdc.mohfw.gov.in/wp-content/uploads/2024/04/guideline_hep20158117187417.pdf.

[11] Jardim RN, Gonçales NS, Pereira JS, Fais VC, Gonçales Junior FL (2008). Occult hepatitis B virus infection in immunocompromised patients. Braz J Infect Dis.

[12] Alkhalifah RH, Alhaddad MJ, Alhashem AT (2023). Prevalence of hepatitis B virus, hepatitis C virus, and HIV infections in hemodialysis patients at Kano Kidney Center. Cureus.

[13] Ersoy O, Yilmaz R, Arici M, Turgan C, Bayraktar Y (2008). Prevalence of occult hepatitis B infection in hemodialysis patients. Dialysis Transplant.

[14] Jadoul M, Bieber BA, Martin P (2019). Prevalence, incidence, and risk factors for hepatitis C virus infection in hemodialysis patients. Kidney Int.

[15] Dharmesti NW, Wibawa ID, Kandarini Y (2022). Hepatitis C seroconversion remains high among patients with regular hemodialysis: study of associated risk factors. Int J Hepatol.

[16] Anees M, Sarwar N, Ahmad S, Elahii I, Mateen FE (2021). Factors associated with seroconversion of hepatitis C virus in end stage renal disease patients. J Coll Physicians Surg Pak.

[17] Hajji M, Barbouch S, Manaa R (2021). National epidemiological study about hepatitis C virus infection among dialysis patients. Saudi J Kidney Dis Transpl.

[18] Masoodi I, Singh C, Wani IA, Wani MM, Ahmed TI, Sheikh RY (2019). Sero conversion of viral hepatitis among end stage renal disease patients on hemodialysis in Kashmir: results of a prospective study. Open Access Maced J Med Sci.

